# The Impact of Variant Philadelphia Chromosome Translocations on the Clinical Course of Chronic Myeloid Leukemia

**DOI:** 10.4274/tjh.2015.0237

**Published:** 2016-02-17

**Authors:** Damla Eyüpoğlu, Süreyya Bozkurt, İbrahim Haznedaroğlu, Yahya Büyükaşık, Deniz Güven

**Affiliations:** 1 Hacettepe University Faculty of Medicine, Department of Internal Medicine, Ankara, Turkey; 2 Hacettepe University Faculty of Medicine Cancer Institute, Basic Oncology, Ankara, Turkey; 3 Hacettepe University Faculty of Medicine, Division of Hematology, Ankara, Turkey

**Keywords:** Chronic myeloid leukemia, Variant Philadelphia, Tyrosine kinase inhibitors, prognosis

## Abstract

Chronic myeloid leukemia (CML) is genetically characterized by the presence of the reciprocal translocation t(9;22) with the formation of Philadelphia (Ph) chromosome. Sometimes, the Ph translocation is generated by variant rearrangements. The prognostic impact of the variant translocations is still controversial. Among the 180 patients with Ph-positive CML who were treated in Hacettepe University Faculty of Medicine Division of Hematology, variant translocations were detected, and retrospectively clinical and prognostic features were described. Also we performed a comprehensive literature review on the prognosis of such variant cases before and after tyrosine kinase inhibitor era. Five patients (2.7%) had variant Ph chromosomes, involved in the rearrangements were chromosomes 2 (2 cases), 11, 14 and 15. Patients were treated with imatinib or dasatinib. All patients reached a stable major molecular response suggesting a prognosis not worse than standard translocation individuals. Our present data were compatible with the data of previous studies indicating no difference in the prognosis between standard and variant translocations in tyrosine kinase inhibitors era of CML.

## Abstract (tr)

Kronik myeloid lösemi (KML) Philadelphia (Ph) kromozomu olarak anılan t(9;22) resiprokal translokasyonuyla karakterize bir hastalıktır. Bazen Ph kromozomu varyant rearranjmanlarla ortaya çıkabilir. Varyant translokasyonların prognostik etkisi halen tartışmalıdır. Hacettepe Üniversitesi Tıp Fakültesi Hematoloji Kliniği’nde tedavi edilen 180 KML hastasından tanı anında varyant translokasyon taşıyanlar tespit edildi, klinik ve prognostik özellikleri retrospektif olarak incelendi. Ayrıca varyant olgularda tirozin kinaz inhibitörleri dönemi öncesi ve sonrasındaki prognoz üzerine geniş bir literatür taraması yapıldı. Hastalardan 5’i (%2,7) tanı anında 2. (2 olgu), 11., 14. veya 15. kromozom rearranjmanlarını içeren varyant Ph taşımaktaydı. Hastalar imatinib veya dasatinib ile tedavi edildi. Hastaların tümünde stabil bir majör moleküler yanıt elde edilmesi standart translokasyona göre daha kötü bir prognoza sahip olmadığını telkin etmektedir. Mevcut verilerimiz, daha önce yapılmış, tirozin kinaz inhibitörleri döneminde standart ve varyant translokasyonlar arasında prognoz açısından farklılık belirtmeyen çalışmalarla uyumluluk göstermektedir.

## INTRODUCTION

Chronic myeloid leukemia (CML) is a proliferative disorder of hematopoietic pluripotent stem cells [1]. It presents with an estimated incidence of 1/100,000 cases per year, which accounts for 15%-20% of all leukemia cases [2]. CML is genetically characterized by the presence of the reciprocal translocation t(9;22) with the formation of the Philadelphia (Ph) chromosome [3]. The BCR-ABL fusion gene encodes a constitutively active protein tyrosine kinase and it is responsible for the leukemia phenotype through the constitutive activation of multiple signaling pathways [4]. The Ph chromosome is detected in around 90% of CML patients, among whom 5%-10% may have variant types [5]. Variant Ph chromosomes can present a simple form (involving 22q11 and one additional breakpoint) or a complex form (involving 22q11, 9q34, and at least one additional breakpoint) [6].

The aim of this study is to assess the frequency and prognosis of CML with variant Ph chromosomes. We also performed a comprehensive literature review to understand the prognosis of such cases before and after the tyrosine kinase inhibitor (TKI) era.

## MATERIALS AND METHODS

### Study Population

Between 2008 and 2014, 180 patients were diagnosed with CML at our institution. The diagnosis of CML was established on the basis of bone marrow examination and supported by cytogenetic and molecular studies. Clinical, cytogenetic, and molecular responses to TKIs were rated according to the European Leukemia Net (ELN) 2013 guidelines [7].

### Cytogenetic Studies

Conventional cytogenetic analysis was performed on unstimulated bone marrow specimens after 24 h of culture. Briefly, the cells were cultured and processed by conventional methods. After trypsin-Giemsa banding (GTG-banding), 20 metaphases were analyzed and karyotypes were interpreted according to the 2013 International System for Human Cytogenetic Nomenclature [8].

## RESULTS

**Figure 1:**
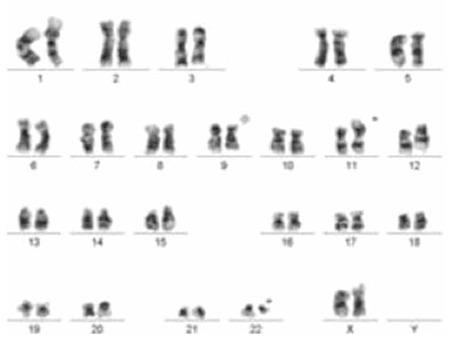
The karyotype of case 3; 46,XX t(9;11;22)(q34;p15;q11.2).

Among the 180 patients with Ph-positive CML, 5 had variant Ph chromosomes. Rearrangements involving chromosomes 2 (2 cases), 11, 14, and 15 were detected. Four patients were female, the median age was 60 (range: 49-68) years, and the median white blood cell count was 64x103/µL (24-177x103/µL). In regard to cytogenetic characteristics, all of the variant Ph translocations were reciprocal three-way translocations that presented at diagnosis (Figure 1). One patient’s follow-up data (case 2) were not available. The other four patients’ median follow-up time was 38.5 months (8-65 months), and TKIs (imatinib, and dasatinib in the case of imatinib failure) were used as therapeutic agents. The main clinical parameters and cytogenetic responses are outlined in Table 1.

For evaluating the literature data on the impact of the variant translocations on the prognosis and clinical features, we performed an English literature review. For this review, the PubMed (http://www.ncbi.nlm.nih.gov/pubmed) and Web of Science (Web of Knowledge [v5.12], Thomson Reuters, http://apps.webofknowledge.com/) databases were used. “CML AND variant philadelphia” and “CML AND variant translocation” were used as keywords. We analyzed the studies in which at least 4 cases were included and TKIs had been used as therapeutics. The literature review was conducted in May 2015. The main criteria of these studies are outlined in Table 2.

## DISCUSSION

In 2%-10% of cases, the Ph translocation is generated by variant rearrangements, involving 9q34, 22q11, and one or several other genomic regions [3]. In our study, 2.7% of our patients exhibited variant Ph chromosomes, which corresponds to the lower margin of the reported range [2,5,9]. Rearrangements involving chromosomes 2 (2 cases), 11, 14, and 15 were detected in our patients. The profile of the variant translocations was similar with those of previous reports [2,10,11]. In our study, 2 out of 3 patients who had been followed for >12 months attained complete cytogenetic response (CCyR) at 12 months. All of the 4 patients for whom follow-up data were available reached major molecular response (MMR) and they were still in MMR at the last follow-up. These data do not suggest worse prognosis compared to our standard Ph patients, which has been reported before [12].

The prognostic impact of the variant translocations was reported in many studies. However, some authors have stated that the involvement of additional oncogenes could be associated with poorer prognosis [10,13,14], while the majority of related studies have confirmed no difference in the prognosis between standard and variant translocations [2,5,9,15,16,17]. The ELN recommendations do not provide any specific advice for patients with variant translocations [7].

Johansson et al. [3] mentioned that the prognostic impact of variant translocations and secondary abnormalities was heterogeneous and most likely related to several parameters, such as time of appearance, specific abnormalities, and treatment modalities.

In the first such comprehensive study, El-Zimaity et al. [9] investigated the characteristics and outcomes of 44 patients with variant translocations among 721 CML patients treated with imatinib. The only significant difference in clinical characteristics was a higher frequency of accelerated phase in those with variant translocations (56% vs. 38%).

In a large retrospective study, Fabarius et al. [16] mentioned that there was no significant difference in the median time of CCyR (0.95 and 1.01 years), the median time to MMR (1.58 and 1.40 years), the 5-year progression-free survival (81% and 90%), and the 5-year overall survival (87% and 92%). In the study of Marzocchi et al. [5], no significant differences in complete hematological response (93% and 98%), CCyR at 12 months (70% and 78%), or MMR at 12 months (57% and 59%) were observed between both groups in terms of the initial therapy with imatinib mesylate.

Hsiao et al. [17] compared the clinical features of CML patients with standard and variant translocations. Apart from the other studies, they not only included TKIs as therapeutic agents but also investigated clinical outcomes of the cytotoxic protocols. It was stated that there was no significant difference in sex, age, complete blood counts, disease status, and survival between variant and classical Ph groups.

On the other hand, several studies pointed out the poor prognosis of variant translocations. Lee et al. [14] stated that variant Ph at diagnosis was associated with lower event-free survival (EFS) (p=0.02) and failure-free survival (p=0.008). Stagno et al. [10] identified that the median amount of BCR-ABL at diagnosis was significantly higher in the variant Ph group. After 18 months of imatinib (8 patients) or nilotinib (2 patients) treatment, 8 patients achieved suboptimal response or failed, while 7 patients had a cytogenetic or a molecular suboptimal response. As a result, the authors stated that complex variant translocations are associated with genomic instability and a more aggressive form of CML. Gorusu et al. [13] confirmed that deletions of the ABL1 or BCL locus were more prevalent in variant translocation CML cases and indicated statistically worse therapeutic responses (p<0.04) and outcomes.

The impact of the variant translocations on Sokal and Euro scores was also found to differ in several studies. Some studies mentioned that there was no significant difference between patients with variant and standard Ph translocations regarding Sokal and Euro scores [2,5,9,16]. On the other hand, it was reported that intermediate Sokal risk score is more frequent in patients with variant translocations and that patients with intermediate Sokal risk had lower EFS (p=0.047) in another study [14].

## CONCLUSION

Between 2% and 10% of patients with CML may have variant translocations [5]. Although it is generally accepted that the clinical, prognostic, and hematological features of CML cases with variant translocations are not distinct from those with the typical t(9;22) translocation [3,18], controversies were found in respect to the prognostic meaning of variant Ph chromosomes [19,20]. Although our patient number was relatively limited, our data were coherent with the studies mentioning no difference in the prognosis between standard and variant translocations in the TKI era.

## Figures and Tables

**Table 1:**
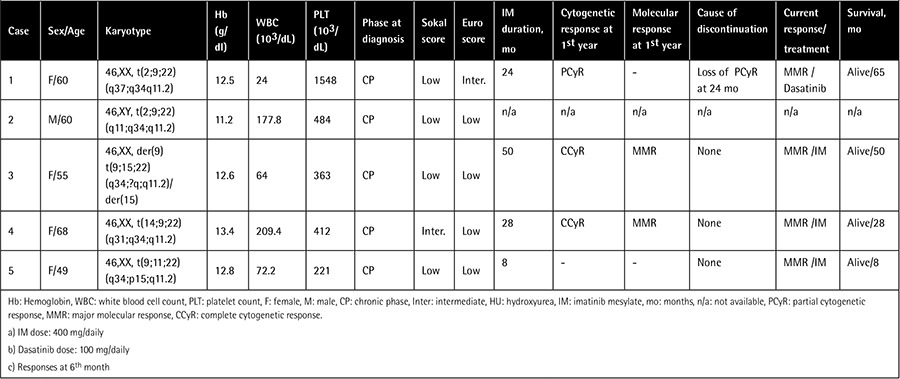
Main clinical, hematological, and cytogenetic characteristics of the patients.

**Table 2:**
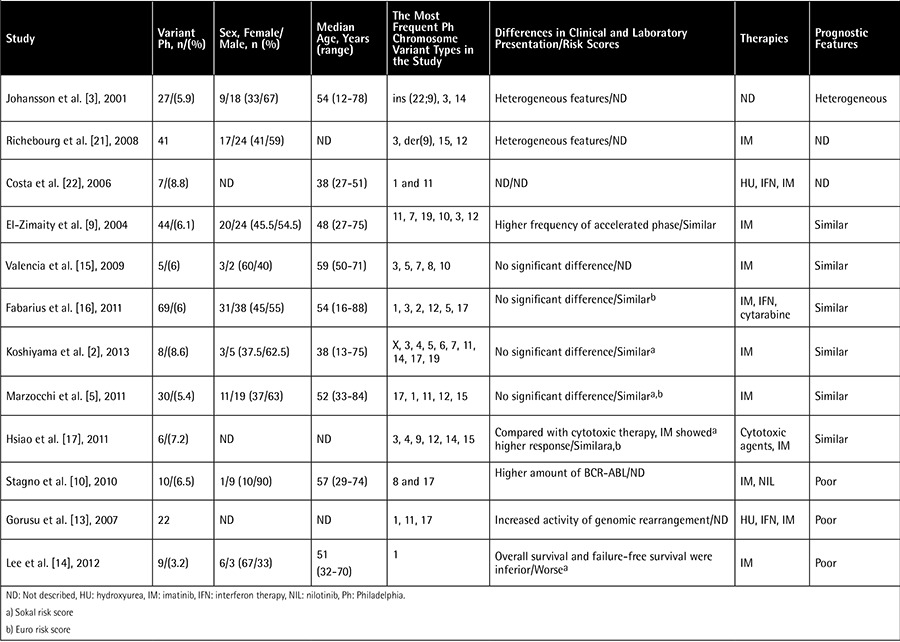
Summary of the studies describing the role of variant Philadelphia in chronic myeloid leukemia patients.
